# Organizing genome engineering for the gigabase scale

**DOI:** 10.1038/s41467-020-14314-z

**Published:** 2020-02-04

**Authors:** Bryan A. Bartley, Jacob Beal, Jonathan R. Karr, Elizabeth A. Strychalski

**Affiliations:** 1 Raytheon BBN Technologies, Cambridge, MA 02138 USA; 2 Icahn Institute and Department of Genetics and Genomic Sciences, Icahn School of Medicine at Mount Sinai, New York, NY 10128 USA; 3 National Institute of Standards and Technology, Gaithersburg, MD 20899 USA

**Keywords:** Synthetic biology, Genomic engineering

## Abstract

Genome-scale engineering holds great potential to impact science, industry, medicine, and society, and recent improvements in DNA synthesis have enabled the manipulation of megabase genomes. However, coordinating and integrating the workflows and large teams necessary for gigabase genome engineering remains a considerable challenge. We examine this issue and recommend a path forward by: 1) adopting and extending existing representations for designs, assembly plans, samples, data, and workflows; 2) developing new technologies for data curation and quality control; 3) conducting fundamental research on genome-scale modeling and design; and 4) developing new legal and contractual infrastructure to facilitate collaboration.

## Abstract (web summary)

Genome-scale engineering requires the integration of a wide range of in silico and in vivo technologies, as well data management procedures and legal infrastructure. Here the authors provide a list of recommendations to address these challenges.

## Introduction

**Fig. 1:**
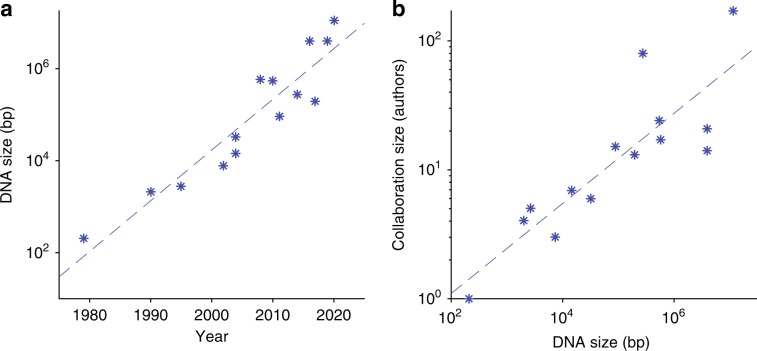
As capabilities for genome engineering have advanced rapidly, the size of teams involved in each pioneering genome engineering project has also increased. **a** From 1980 to present, the size of the largest engineered genomes has grown exponentially, doubling approximately every 3 years. This trend suggests that gigabase engineering could become feasible by 2050. **b** The number of authors credited with producing these genomes has also grown exponentially. This trend suggests that engineering gigabase genomes will require the effort of ~500 individuals—either directly as part of a team or indirectly through an ecosystem of tools, services, automation, and other resources. The data for this figure are provided in Table 1.

Engineering the entire genome of an organism promises to enable large-scale changes to its organization, function, and interactions with its environment, with broad potential for impacts across science, industry, medicine, and society^1^. The past several decades have seen remarkable progress in our capability to synthesize DNA and modify genomes^2–4^. Since Khorana created the first synthetic gene 40 years ago^5^, our capability to construct DNA sequences has doubled, approximately every 3 years (Fig. 1a), progressing from plasmids in the early 1990’s^6,7^, viruses in the early 2000’s^8^, and gene clusters in the mid-2000’s^9,10^, to the first bacterial chromosome in 2008^11,12^. Recently, several groups have re-engineered the 4 Mb genomes of *Escherichia coli*^13,14^ and *Salmonella typhimurium*^15^, and the Synthetic Yeast (Sc 2.0) project^16,17^ has nearly completed re-engineering an 11.4 Mb genome for *Saccharomyces cerevesiae*^18^. Looking ahead, in 2016 leaders from academia and industry formed Genome Project-Write^1^ to initiate the engineering of the gigabase genomes of higher-order eukaryotes. The goals of the GP-Write consortium include engineering a virus-resistant, ultra-safe human-derived cell line for pharmaceutical production^19^.

## From engineering genes to engineering genomes

Moving to the gigabase scale poses major technological and scientific challenges. Challenges related to DNA synthesis and editing have been discussed extensively in the literature^20–23^. Significant attention has also been devoted to the challenges of modeling^24,25^, designing^17,26,27^, and testing^28^ genomes. Less attention, however, has been devoted to the technologies, repositories, standards, and other resources needed to integrate these tasks into a cohesive workflow.

We contend that workflow integration is a first-class problem for gigabase-scale genome engineering. Over the last 40 years, the number of authors of pioneering genome engineering projects has risen markedly with genome size, suggesting that the complexity of genome engineering is also scaling with the size of the genome (Fig. 1b). If these trends continue, engineering a gigabase genome would be projected to become possible in ~2050 and require a team with the capabilities of around 500 investigators. To manage projects of such complexity without massive teams, we advocate for the development of an ecosystem of tools, services, automation, and other resources, which could enable a modestly sized team of bioengineers to indirectly access the equivalent capabilities of hundreds of people. To this end, we have examined the emerging design–build–test–learn workflow for genome engineering, identifying key interfaces and making recommendations for the adoption or development of technologies, repositories, standards, and frameworks.

**Table 1:** Year, genome size (bp), and the number of authors involved in pioneering genome engineering projects of the last 30 years.

Year	DNA size (bp)	Collaboration size (# authors)	Reference	Notes
1979	207	1	Khorana^5^	First synthetic gene
1990	2050	4	Mandecki et al.^6^	First synthetic plasmid
1995	2700	5	Stemmer et al.^7^	Synthetic plasmid
2002	7.5000E+03	3	Cello et al.^8^	Polio virus cDNA
2004	1.4600E+04	7	Tian et al.^9^	rRNA genes
2004	3.1656E+04	6	Kodumal et al.^10^	Gene cluster
2008	5.8297E+05	17	Gibson et al.^11^	Mycoplasma genitalium
2010	5.3100E+05	24	Gibson et al.^12^	Mycoplasma mycoide, JCVI synthetic cell
2011	9.1010E+04	15	Dymond et al.^16^	Sc 2.0 synIXR
2014	2.7287E+05	80	Annaluru et al.^17^	Yeast chromosome synIII
2016	3.9700E+06	21	Ostrov et al.^13^	Partially recoded *E. coli*, 62 K edits in genome
2017	2.0000E+05	13	Lau et al.^15^	Salmonella typhimurium partial genome
2019	4.0000E+06	14	Fredens et al.^14^	Recoded *E. coli*
2020	1.14E+07	172	Richardson et al.^18^	Sc 2.0 estimated completion date; Genome size from Table 3 in reference; Collaboration size estimated from Sc 2.0 website

These data are plotted in Fig. 1

## An emerging workflow for genome engineering

**Fig. 2:**
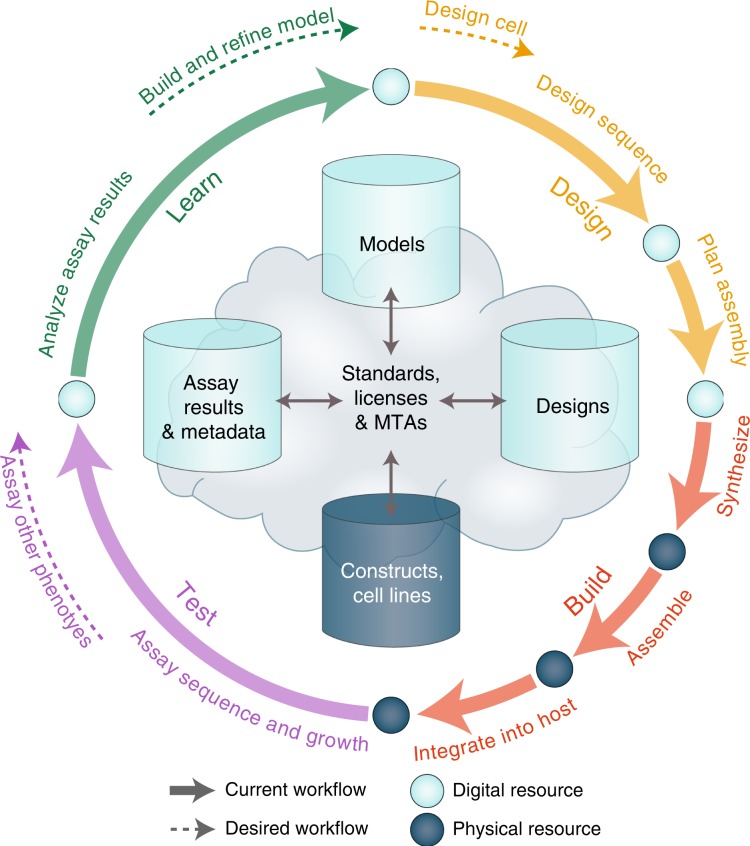
The emerging design–build–test–learn workflow for genome engineering is shown schematically with current (solid arrows) and likely future (dashed arrows) tasks, interfaces (circles), and repositories (cylinders), either digital (light) or physical (dark).

Recently, a number of groups have proposed or developed workflows for organism engineering^3,18,27–32^, converging toward a common engineering cycle consisting of the four stages shown in Fig. 2. These stages are (1) Design**:** bioengineers use models and design heuristics to specify a genome with an intended phenotype; (2) Build**:** genetic engineers construct the desired DNA sequence in a target organism; (3) Test**:** experimentalists assay molecular and behavioral phenotypes of the engineered organism; (4) Learn**:** modelers analyze the discrepancies between the desired and observed phenotypes to develop improved models and design heuristics. The process is repeated until an organism with the desired phenotype is identified. This incremental approach enables engineering despite our incomplete understanding of the complexities of biology.

The inner loop in Fig. 2 indicates the workflow used by many current genome engineering projects, which have primarily focused on “top-down” refactoring of existing genomes, e.g., by rewriting codons or reducing genomes to essential sequences. In the longer term, one of the key aims of synthetic biology is to engineer organisms that have novel phenotypes by “bottom-up” assembly of modular parts and devices^33^. At a much smaller scale, organism engineers are already beginning to use this approach to engineer novel metabolic pathways for commercial production of high-value chemicals^34–36^. For gigabase genome engineering, this approach will likely require more complex workflows that utilize more sophisticated design tools, phenotypic assays, data analytics, and models (outer loop of Fig. 2).

Executing these multistep workflows requires exchanging a wide range of materials, information, and other resources between numerous tools, people, institutions, and repositories. The design phase must communicate genome designs to the build phase, the build phase must deliver DNA constructs and cell lines to the test phase, the test phase must transmit measurements to the learn phase, the learn phase must provide models and design heuristics to the design phase, and workflows must be applied to coordinate the interaction and execution of tools across all of these stages.

In addition to these technical challenges, genome engineering must also address a number of safety, security, legal, contractual, and ethical issues. Throughout genome engineering workflows, bioengineers must pay careful attention to biosafety, biosecurity, and cybersecurity. To execute genome engineering workflows across multiple institutions, bioengineers must navigate materials transfer agreements, copyrights, patents, and licenses.

Every aspect of this genome engineering workflow must be scaled up to handle gigabase genomes. Ultimately, much or all of each step should be automated, and each interface between steps should be formalized to facilitate machine reasoning, removing the ad hoc and human-centric aspects of genome engineering as much as possible. In many cases, this can be facilitated by adopting or extending solutions from smaller-scale genome engineering, as well as solutions from related fields such as systems biology, genomics, genetics, bioinformatics, software engineering, database engineering, and high-performance computing. Other challenges of gigabase genome engineering, however, are likely to require the development of novel systems or additional fundamental research.

## Identifying and closing gaps in the state of the art

In this section, we discuss the integration challenges identified in the previous section, reviewing the state of the art in technologies and standards with respect to the emerging needs of gigabase genome engineering. Instead of focusing on specific evolving protocols and methods, which are likely to advance rapidly, we consider the information that must be communicated to enable protocols or methods to be composed into a comprehensive workflow. Through this analysis, we identify critical gaps and opportunities, where additional technologies and standards would facilitate workflows that can effectively deliver gigabase engineered genomes. Table 2 summarizes the potential solutions that we have identified, which are detailed in the following subsections.

**Table 2:** Potential approaches for integrating the emerging gigabase engineering workflow, labeled for reference.

For each interface in the emerging workflow, our recommendations fall into one of three categories: adopt or extend relatively mature existing methods (green), develop new solutions or expand nascent methods (yellow), and conduct additional fundamental research (red)

## Genome refactoring and design

Current genome engineering projects have focused primarily on refactoring genomes while preserving their cellular function. For example, three recent projects have involved eliminating nonessential elements^27^, reordering genes^17^, and inserting metabolic pathways^37^. At this level, two critical challenges for scaling are accessing well-annotated source genomes and representing and exchanging designs for modified genomes. More complex changes of organism function will pose additional challenges related to composing parts to produce novel cellular functions.

Currently, genome design generally involves modifying pre-existing organism sequences, such as those available in the public archives of the International Nucleotide Sequence Database Collaboration (INSDC)^38^, which currently contains ~$1{0}^{5}$ bacterial genomes and hundreds of eukaryotic genomes^39–43^. Functional annotation is key, as genome engineers will need to consider tissue-specific expression patterns, regulatory elements, structural elements, replication origins, clinically significant sites of DNA recombination and instability, etc. The consistency of annotations is a key challenge, as many genomes have been annotated by different toolchains that produce significantly different annotations. For example, the human reference genomes generated by the RefSeq and GENCODE projects have notable differences^44,45^ with likely engineering consequences, such as ability to predict loss-of-function from interaction with alternative splicings. Much of this knowledge is also dispersed among different resources, though annotations can be integrated with the aid of services such as NCBI Genome Viewer^46^, WebGestalt^47^, and DAVID^48^. For moving to the gigabase scale, improved annotation APIs will be valuable, as would estimates of the confidence and reliability of annotations, such as the RefSeq database does with the Evidence and Conclusion Ontology^49^.

The gigabase scale poses challenges for the representation and exchange of genome designs as well. Common formats such as GenBank and EMBL are monolithic in their treatment of sequences, which makes it difficult to integrate or harmonize editing across multiple concurrent users, and can even cause difficulties in simply transferring the data. Two formats better suited for genome engineering are the Generic Feature Format (GFF) version 3 and the Synthetic Biology Open Language (SBOL) version 2^50^. GFF3 allows hierarchical organization of sequence descriptions (e.g., genes may be organized into clusters, and clusters into chromosomes), uses the Sequence Ontology^51^ to annotated sequences, and has already been used in the Sc 2.0 genome engineering project^18^. SBOL 2 is also routinely used for hierarchical description of edited genomes^52^ and can interoperate with GFF3 (though GFF3 only represents a subset of SBOL)^53^. SBOL provides a richer design-centric language, including support for variants, libraries, and partial designs (e.g., identifying genes in a cluster, but not yet particular variants or cluster arrangement), other elements and cellular functions (e.g., proteins, metabolic pathways, regulatory interactions). SBOL also interoperates with models encoded in the Systems Biology Markup Language (SBML)^54,55^. Both GFF3 and SBOL, however, would benefit from more stable specifications of sequence positions within chromosomes, as sequence index is fragile to changes and sequence uncertainties. SBOL supports (and GFF3 could be extended to support) expression of nonstandard bases and sequence modifications in an enhanced sequence encoding language such as BpForms^56^.

Representations of genome designs also need to express design constraints and policies, such as removal of restriction sites, separation of overlapping features, replacement of codons, and optimization for DNA synthesis. Projects such as Sc 2.0 have implemented this with a combination of guidelines for human hand-editing and custom software tools, and DNA synthesis providers provide interfaces to check for manufacturability constraints. At the gigabase scale, however, it will be beneficial to adopt more powerful and expressive languages for describing design policies, such as rule-based ontologies^57,58^, and to include assembly and transformation plans in design representations to simplify adjustments for manufacturability. JGI’s BOOST tool provides a prototype in this direction^59^. SBOL is well-suited for this task, though GenBank and GFF3 could also, at least in principle, be extended to encode such information.

Modeling will become increasingly important as genome engineering moves beyond refactoring and recoding into more complex changes to an organism’s function. Genome-scale metabolic models^60,61^ and whole-cell models^62^ can be constructed by combining biochemical and genomic information from multiple databases, such as BioCyc^63^ and the SEED^64^. Models will also need to predict the behavior of organisms that are composed of separately characterized genetic parts, devices, pathways, and genome fragments. Substantial fundamental research still needs to be conducted to make such models practical at the gigabase scale.

## Building engineered genomes

Technology and protocols for building engineered genomes are advancing rapidly, with potential paths to the gigabase scale discussed, for example, in ref. ^1^ and ref. ^23^. Depending on the specific host and intended function of the engineered organism, there are numerous potential approaches and protocols for DNA synthesis, assembly, and delivery. Currently, there is an unmet need for guidance on best practices for measuring, tracking, and sharing information regarding engineered genomes and intermediate samples.

Manipulating DNA during assembly offers ample opportunities for reduced yield, breakage, error, and other sources of uncertainty in achieving the designed DNA sequence. Protocols and commercial kits to assemble shorter DNA fragments into larger constructs often involve amplification, handling, purification, transformation, or other storage and delivery steps that can increase uncertainty in the quality and quantity of the DNA. Assembled DNA may also include added sequences that are not biologically active, as in the case for some methods using restriction enzymes, or scars, such as occur may occur with Golden Gate Assembly^65^ or MoClo^66^. Gibson Assembly^67^ is scarless, but the yield and specific results may depend on the secondary structure of the DNA fragments. Thus, in addition to sequence information, workflows will likely need extended representations that can also track the full range of information likely to affect assembly products, including DNA secondary structure, assembly method, sequences required for assembly and their location along the DNA molecule (e.g., landing pads or sequences for compatibility with protocol-hosting strains of *E. coli* or yeast), and intended epigenetic modifications. The results verifying both intermediate and final sequence onstruction are typically produced in the FASTQ format^68^, which is generally sufficient for smaller constructs. To operate on large-scale genomes, however, more comprehensive descriptions of a genome and its variations may be made with representations such as GVF^69^ or SBOL^70^.

Suitable options for the delivery of large, assembled DNA constructs and whole genomes are generally lacking. The yield of existing processes, such as electrical and chemical transformation or genome transplantation, could be improved significantly to increase their utility, and a broader range of approaches should be developed for use with any organism and cell type. This may also require identifying new cell-free environments or cell-based chassis for assembling and manipulating DNA that also have compatibility with genome packaging and delivery systems into host organisms. To facilitate such development, delivery protocols and their associated information regarding number of biological and technical replicate experiments, methods, measurements, etc. should be available in a machine-readable format. This should include information regarding the host cell, such as its genotype, which is often not fully verified. The adoption of best practices from industrial biomanufacturing settings and implementation of laboratory information management systems (LIMS) could provide a path forward toward integrating appropriate measurements, process controls, and information handling, as well as the tracking and exchange of samples. Advancing the use of automation to support the build step of the genome engineering workflow requires evaluating which steps may reduce costs and speed results, the availability of automated methods, ways to effectively share those methods and adapt them across platforms and manufacturers, and ways to more simply integrate and tune automated workflows.

## Testing the function of engineered genomes

Strain fitness and other phenotypes can be assessed via a wide range of biochemical and omics measurements, the details of which are beyond the scope of this discussion. In all cases, however, collaborating organizations will need to agree on specific measurements, along with control and calibration measurements, to ensure that the results can be compared and used across the participating laboratories.

DNA constructs are often evaluated for their associated growth phenotypes to determine the nature and extent of unexpected consequences for cell function and fitness due to the revised genome sequence. Engineered cell lines should also be evaluated for robustness to changes in the environmental context that the cells are likely to experience during typical use in the intended application, as well as stability over relevant timescales to evolution or adaptation. This is complicated by the need for shared definitions and measurements for fitness, metabolic burden, and other phenotypic properties.

Standard protocols, reference cell lines, and the use of experimental design are examples of tools available to increase the rigor and confidence in conclusions that can be drawn from testing. It will likely also be useful to develop standards and measurement assurance for testing engineered genomes. Such foundations can be used to help identify relationships between genotype and phenotype or determine the contributions of biological stochasticity and measurement uncertainty to the overall variability in a measured trait, though comprehensive methods of this sort are likely to require significant fundamental research.

Calibration of biological assays aids in comparing results both within a single laboratory and across different laboratories. Recent studies, for example, for fluorescence^71,72^, absorbance^73^, and RNAseq^74^ measurements, demonstrate the possibility of realizing scalable and cost-effective comparability in biological measurements. Organism engineering is likely to be facilitated by the development of additional calibrated measurement methods and absolute quantitation of an organism’s properties.

Establishing shared representations and practices for metadata, process controls, and calibration will also be critical. Automation-assisted integration and comparison of the data, metadata, process controls, and calibration across laboratories will facilitate both the testing process and learning through modeling and simulation. Some existing ontologies can be leveraged for this purpose, such as the Experimental Conditions Ontology^75^ (ECO), the Experimental Factor Ontology^76^ (EFO), and the Measurement Method Ontology^75^ (MMO). In addition, appropriate LIMS tooling and curation assistance software (e.g., RightField^77^) will be vital for enabling such metadata to be created consistently, correctly, and in a timely fashion, by limiting the required input from human investigators.

## Learning systematically from test results

As genome engineering affects systems throughout an organism, comprehensive models are needed that can help to both predict and interpret the relationship between genotype and phenotype. Although some models have been constructed for a whole cell^62^ or whole organism^78^, developing and tuning such models is extremely challenging. To scale to gigabase genomes, it will be valuable develop improved capabilities for creating, calibrating, and verifying models.

The first challenge in learning from the data is discovering and marshaling the data needed. Partial solutions exist, such as the workflow model introduced in SBOL 2.2^50^, and ontologies such as the Open Biological and Biomedical Ontology^79^, the Experimental Factor Ontology^76^, the Systems Biology Ontology^80^, and phenotype ontologies^81,82^. These will need to be integrated and extended to cover the full range of needs for genome engineering.

Automation-assisted generation and verification of models at scale, however, still have many open fundamental research challenges, including addressing the combinatorial complexity of biology and the multiple scales between genomes and organismal behavior, high-performance simulation of large models, model verification, and representation of model semantic meaning and provenance^24,25^.

Until we have comprehensive predictive models, engineers will likely rely on ad hoc combinations of predictive models of parts of organisms, data-driven models, and heuristic design rules. For example, constraint-based models are often used in metabolic engineering^34^, PSORTb^83^ can be used to help target proteins to specific compartments, and GC-content optimization can be used to improve host compatibility^84^. Gigabase-scale genome engineering will require applying many such models simultaneously, and thus will benefit from adopting existing standard formats designed to facilitate biological model sharing and composition, such as SBML^85^, CellML^86^, NeuroML^87^, and other standards in the Computational Modeling in Biology Network (COMBINE)^88^. Large numbers of models in these formats can already be found in public databases, such as BioModels^89^, the NeuroML database^90^, Open Source Brain^91^, and the Physiome Model Repository^92^. Similarly, repositories such as Kipoi^93^ and the DockerHub repository^94^ can already be used to share data-driven models. Further extensions to such formats, however, will be valuable for automating the learning process, including associating semantic meaning with model components, capturing the provenance of model elements (e.g., data sources, assumptions, and design motivations), and capturing information about their predictive capabilities and applicable scope.

To increase automation in learning such models from data, it will likely be valuable to develop new repositories of models of individual biological parts that can be composed into models of entire organisms^95,96^; new methods for generating model variants that explain new observations by incorporating models of additional parts, alternative kinetic laws, or alternative parameter values; and new model selection techniques for nonlinear multiscale models^97^.

## Coordination and sharing in complex workflows

Tasks in isolation are not enough: efficient operation of the design–build–test–learn cycle for engineering gigabase genomes will require coordinating all of the numerous heterogeneous tasks discussed into clear, cohesive, reproducible workflows^98,99^ for software interactions, for laboratory protocols, and for management of tasks and personnel. Automating workflows also provides opportunities to implement best practices for cybersecurity, cyberbiosecurity, and biosecurity.

For integrating informational tasks, computational workflow engines enable specification, reproducible execution, and exchange of complex workflows involving multiple software programs and computing environments. Current workflow tools include both general tools, such as the Common Workflow Language (CWL)^100^, the Dockstore^101^ and MyExperiment^102^ sharing environments, and the PROV ontology for tracking information provenance^103^ (which is already being applied to link design–build–test–learn cycles in SBOL^50^). There are also a number of bioinformatics-focused engines, including Cromwell^104^, Galaxy^105^, NextFlow^106^, and Toil^107^. These can be readily adopted for gigabase engineering through steps such as including CWL files in COMBINE archives^108^, developing REST or other programmatic interfaces for databases used in genome engineering, containerization^109^ of genome engineering computational tools, and depositing these containers to a registry such as DockerHub^94^. Other enhancements likely to be useful include the development of graphical workflow tools for genome engineering, an ontology for annotating the semantic meaning of workflow tasks, and the application of issue tracking systems, such as GitHub issues^110^ or Jira^111^, to help coordinate teams on the complex tasks involved in designing genomes that require human intervention.

For experimental protocols, a number of technologies have already been developed to automate and integrate experimental workflows as well. Laboratory automation systems can greatly improve both reproducibility and efficiency^112^ and can also be integrated with LIMS^113^ to help track workflows and reagent stocks. A number of automation languages and systems have been developed, including Aquarium^114^, Antha^115^, and Autoprotocol^116^. Although these have not been widely adopted, they have been successfully applied to genetic engineering (e.g., ref. ^117^), and gigascale genome engineering would benefit from standardization and integration of such systems for application to build and test protocols.

Once links are established across different portions of a workflow, unified access to information in databases for various institutions and stages of the workflow can be accomplished using standard federation methods and any of the various mature open tools for database management systems (DBMS). Scalable sharing would be further enhanced by adoption of the FAIR (findable, accessible, interoperable, reproducible) data management principles^118^, which puts specific emphasis on automation friendliness of data sharing. Repositories that support these principles and are applicable to genome engineering include FAIRDOMHub^119^, Experimental Data Depot (EDD)^120^, and SynBioHub^121^.

## Contracts, intellectual property, and laws

Large-scale genome engineering also poses novel challenges in coordinating legal and contractual interactions. When using digital information, both humans and machines need to know the accompanying copyright and licensing obligations. Systematic licensing regimes have been developed for software by the Open Source Initiative (OSI) and other software organizations^122^ and for media and other content with the Creative Commons (CC) family of licenses^123^, both of which readily allow either a user or a machine to determine if a digital object can be reused, if its reuse is prohibited, or if more complicated negotiation or determination is required. Such systems can be applied to much of the digital information in genome engineering. Care will need to be taken, however, regarding sensitive personal information and European Union database protection rights, which these do not address.

Transfer of physical biological materials was first standardized in 1995 with NIH’s Uniform Biological Materials Transfer Agreement (UBMTA), which is used extensively by organizations such as Addgene. Broader and more compatible systems have been developed in the form of the Science Commons project^124^ and the OpenMTA^125^. There are still significant open problems regarding compliance with local regulatory and legal systems, however, particularly when materials cross international borders. Moreover, material transfer agreements generally do not address the intellectual property for materials, which is typically governed through patent law. No publicly available system yet supports automation for patent licensing. Development of automation-friendly intellectual property management might be supported by defining tiered levels that are simultaneously intelligible for the common user, legal experts, and computer systems—though establishing which material or usages can be classified into which tiers may be a difficult process of legal interpretation. Effective use in automation-assisted workflows will also require recording information about which inputs are involved in the production of results, using mechanisms such as the PROV ontology^103^.

Finally, organizations will also need to manage the level of exposure of information, whether due to issues of privacy, safety, publication priority, or other similar concerns. Again, no current system exists, but a basis for developing one may be found in the cross-domain information sharing protocols that have been developed in other domains^126,127^.

## Recommendations and outlook

In summary, scaling up to gigabase genomes presents a wide range of challenges (Table 2). We observe that these challenges cluster into four general themes, each with a different set of needs and paths for development.

The first theme is representing and exchanging designs, plans, data, metadata, and knowledge. Managing information for gigabase genome design requires addressing many challenges regarding scale, representation, and standards. Relatively mature technologies exist to address most individual needs, as well as to assist with the integration of workflows. The practical implementation of effective workflows will require significant investment in building infrastructure and tools that adopt these technologies, including domain-specific extensions and refinements.

The second theme is sharing and integrating data quality and experimental measurements. Sharing and integrating information arising from measurements of biological material poses significant challenges. It remains unclear what information would be advantageous to share, given the difficulty of obtaining and interpreting measurements of biological systems and the expense and unfavorable scaling of data curation. However, effective integration depends on associating reproducible measurement data with well-curated knowledge and metadata in compatible representations. A number of potential solutions exist for each of these, but significant investment will be needed to investigate how the state of the art can be extended to address these needs.

The third theme is integration of modeling and design at the gigabase scale. Considerable challenges surround efforts to develop a deeper understanding of the relationship between genotype and phenotype, regarding both the interpretation of experimental data and the application of that data to create and validate models, which may be applied in computer-assisted design. Long-term investment in fundamental research is needed, and the suite of biological systems of varying complexity, from cell-free systems to minimal and synthetic cells to natural living systems, may offer suitable experimental platforms for learning the relationship between genotype and phenotype.

Finally, the fourth theme is technical support for Ethical, Legal, and Societal Implications (ELSI) and Intellectual Property (IP) at scale. At the gigabase scale, computer-assisted workflows will be necessary to manage contracts, intellectual property, materials transfers, and other legal and societal interactions. Such workflows will need to be developed by interdisciplinary teams involving experts in law, ELSI issues, software engineering, and knowledge representation. Moreover, it will be critical to address these issues early, to minimize the potential for problematic entanglements associated with the reuse of resources.

In short, engineering gigabase-scale genomes presents significant challenges that will require coordinated investment to overcome. Because many other areas of bioscience face similar challenges, solutions to these challenges will likely also benefit the broader bioscience community. Importantly, the challenges of scale, integration, and lack of knowledge faced in genome engineering are not fundamentally different in nature than those that have been overcome previously in other engineering ventures, such as aerospace engineering and microchip design, which required organizing humans and sharing information across many institutions over time. Thus, we expect to be able to adapt solutions from these other fields for genome engineering.

Investment in capabilities for genome engineering workflows is critical to move from a world in which genome engineering is a heroic effort to one in which genome engineering is routine, safe, and reliable. Investment in workflows for genome engineering will support and enable a vast number of projects, including many not yet conceived, as was the case for reading the human genome. As workflow technologies improve, we anticipate that the trends of expanding team size will eventually reverse, enabling high-fidelity whole-genome engineering at a modest cost and supporting a wide range of medical and industrial applications.
